# Pediatric Renal Transplantation

**Published:** 2012-05-01

**Authors:** B. Saeed

**Affiliations:** *Pediatric Nephrology Division, Kidney Hospital, Damascus, Syria*

**Keywords:** Transplantation, Kidney, End-stage renal disease, Pediatrics

## Abstract

Although the number of children with end-stage renal disease (ESRD) in need for renal transplantation is small compared with adults, the problem associated with renal transplant in children are numerous, varied, and often peculiar. Pre-emptive transplantation has recently been growing in popularity as it avoids many of the associated long-term complications of ESRD and dialysis. Changes in immunosuppression to more potent agents over the years will have affected transplant outcome; there is also evidence that tacrolimus is more effective than cyclosporine. This review will discuss the short- and long-term complications such as acute and chronic rejection, hypertension, infections, and malignancies as well as factors related to long-term graft function.

Chronic allograft nephropathy is the leading cause of renal allograft loss in pediatric renal transplant recipients. It is likely that it reflects a combination of both immune and nonimmune injury occurring cumulatively over time so that the ultimate solution will rely on several approaches. Transplant and patient survival have shown a steady increase over the years. The major causes of death after transplantation are cardiovascular disease, infection and malignancy. Transplantation in special circumstances such as children with abnormal urinary tracts and children with diseases that have the potential to recur after transplantation will also be discussed in this review. Non-compliance with therapeutic regimen is a difficult problem to deal with and affects patients and families at all ages, but particularly so at adolescence. Growth may be severely impaired in children with ESRD which may result in major consequences on quality of life and self-esteem; a better height attainment at transplantation is recognized as one of the most important factors in final height achievement.

Although pediatric kidney transplantation is active in some parts of many developing countries, it is still inactive in many others and mostly relying on living donors. The lacking deceased programs in most of these countries is one of the main issues to be addressed to adequately respond to organ shortage.

In conclusion, transplantation is currently the best option for children with ESRD. Although improvement in immunosuppression demonstrated excellent results and has led to greater 1-year graft survival rates, chronic graft loss remains relatively unchanged and opportunistic infectious complications remain a problem

## INTRODUCTION

Kidney transplantation is now considered the treatment of choice for end-stage renal disease (ESRD) in children [1], because it is associated with a better quality of life, productivity and growth of children and longer patient survival than what can be achieved by any modality of long-term dialysis [2,3]. Graft survival has improved significantly in recent years, mainly due to improved immunosuppressive strategies [4]. The main problem, however, chronic allograft nephropathy (CAN), remains unresolved. In CAN, specific immunological (also nonimmunological) risk factors seem to play a role [5], so that the ultimate solution to the problem will rely on several approaches.

**CHRONIC KIDNEY DISEASE IN CHILDREN**

In contrast to the increasing availability of information pertaining to the care of children with chronic kidney disease (CKD) from large-scale observational and interventional studies, epidemiological information on the incidence and prevalence of pediatric CKD is currently limited, imprecise, and flawed by methodological differences between the various data sources. There are distinct geographic differences in the reported causes of CKD in children, in part due to environmental, racial, genetic, and cultural (consanguinity) differences. However, a substantial percentage of children develop CKD early in life, with congenital renal disorders such as obstructive uropathy and aplasia, hypoplasia, or dysplasia being responsible for almost one-half of all cases [6]. The most favored ESRD treatment modality in children is renal transplantation, but a lack of health care resources and high patient mortality in the developing world limits the global provision of renal replacement therapy (RRT) and influences patient prevalence [7]. Now most registries report that approximately two-thirds of children and adolescents on ESRD programs have a transplant [8].

**CHILDREN ARE NOT SMALL ADULTS**

Although the number of children with ESRD in need for renal transplantation is small compared with adults, the problem associated with renal transplant in children are numerous, varied, and often peculiar [9]. When compared to adults, children have major medical differences in their response and tolerance to medication and the procedure involved in transplantation. Moreover, children and adolescents with ESRD have unique features that are different from the adult population, including the need to achieve normal growth and normal cognitive and psychological development. Therefore, the experience in adults cannot be extrapolated to children.

**PRE-EMPTIVE TRANSPLANTATION**

Pre-emptive transplantation (PET), which denotes transplantation prior to the initiation of dialysis, has recently been growing in popularity, as it is postulated that transplanting children before they develop symptoms of severe uremia avoids many of the associated long-term complications of ESRD and dialysis [10]. Avoiding dialysis and all of its hazards is one of the most important advantages of PET. Dialysis is regarded by most patients and parents, as an inconvenient experience requiring frequent hospital visits for hemodialysis and frequent dialysate exchanges for peritoneal dialysis. It has been shown that children submitted to PET achieved normal parathyroid hormone levels sooner than dialysis children [11]. Data from the North American Pediatric Renal Transplant Cooperative Study (NAPRTCS) indicate that the most common reason for selecting PET in the United States is basically to avoid dialysis by both parents and children. The lack of satisfactory catch-up growth in most transplanted children emphasizes the importance of transplanting children with chronic renal failure before they reach a stage of severe uremia and require dialysis. Therefore, PET provides a better option for the prevention of short stature with all its co-morbidity and psychosocial implications. As most of the effects on cognitive development are related to uremia, it is postulated that PET will have further favorable effect over post-dialysis transplantation [12]. PET is cost effective. In the United States, it is estimated that Medicare expenditures for children with ESRD range from US$ 14,000 for transplant recipients to US$ 43,000 for dialysis patients per year [13]. Therefore, decreasing the period in which patients are on dialysis or even omitting dialysis altogether whenever appropriate, has a significant effect on the cost of the care of ESRD children.

**IMMUNOSUPPRESSION**

There have been considerable changes in the use of immunosuppression over the years. NAPRTCS has reported that the use of cyclosporine has decreased from 82.3% in 1996 to 20.7% in 2003. In contrast, use of tacrolimus has increased from 5.5% to 67.1% over the same period. There has also been a move away from azathioprine (AZA), from 56.4% to 1.9%, towards mycophenolate mofetil (MMF), use of which is now approximately 57.4%. Antibody induction has also moved away from antithymocyte globulins (ATG) and OKT3 to anti-IL2 receptor blockers. The use of steroid-sparing regimens is also becoming common [14]. Clearly, changes in immunosuppression to more potent agents over the years will have affected transplant outcome. There is evidence that the use of MMF with cyclosporine is associated with better outcome at five years than a similar regimen using AZA; transplant survival was 90.7% for MMF and 68.5% for AZA patients and the cumulative rejection-free survival was also better in the MMF group (51.2%) than AZA patients (37.0%) [15]. The projected half-life in the same study was 14.4 and 4.5 years in patients with rejection, and 18.7 and 4.5 years in those without rejection, in the MMF and AZA treated groups, respectively [15], which could indicate that the absence of rejection would be associated with better outcome. There is also evidence that tacrolimus is more effective than cyclosporine: a randomized trial of steroids and AZA with either tacrolimus or cyclosporine demonstrated that tracrolimus was significantly more effective than cyclosporine in both preventing acute rejection and maintaining graft function, with a 4-year transplant survival rate of 86% and 69%, respectively, and glomerular filtration rate of 71.5 mL/min/1.73 m² and 53.0 mL/min/1.73 m² body surface area, respectively [4].

**ACUTE REJECTION**

Acute rejection is responsible for 13% to 21% of graft failure in children [16,17]. The number, the severity, and the response to corticosteroids of acute allograft rejection episodes during the first six months post-transplantation are a major determinant of long-term graft function and survival [17-19]. However, the use of new immunosuppressive regimens has significantly decreased the rate of initial episodes of rejection [20]. In the late nineties, it has been shown that early acute rejection may also increase the risk of patient death, due to opportunistic infections during aggressive antirejection therapy [21]. However, with the use of newer anti-viral and anti-opportunistic prophylaxis, it is probable that patients being transplanted nowadays are no longer exposed to an increased risk of early acute rejection related death. The risk of acute rejection by the end of the first year post-transplantation is lower with living donor transplantation [20].

**POST-TRANSPLANT HYPERTENSION**

The long-term success of renal transplantation is limited by either the occurrence of chronic allograft nephropathy or by death of the patient. Cardiovascular diseases are amongst the main causes of mortality in patients undergoing kidney transplantation and also play a role in the pathogenesis of chronic allograft nephropathy [22]. Hypertension is common after transplantation, and its incidence varies with time, ranging from 46%, 40%, and 66% of children at 1, 5, and 10 years, respectively [23]. Tejani reported hypertension in 86% of children a month after renal transplantation, and in 100% of those who were hypertensive before transplantation [24]. However, it is worth saying that this paper was reported in 1983 when the only immunosuppression available was high dose steroid and AZA. Many children are presently being transplanted with steroid avoidance or minimal dose of steroid and probably they are not hypertensive or minimally so for a short time. Hypertension has a multifactorial etiology, the significance of each etiological factor being difficult to ascertain [24]. Post-transplant hypertension is a negative factor, both for graft and patient survival. The presence of hypertension is a significant and independent predictor of poor long-term transplant function, regardless of the number of rejection episodes or transplant function at one year [25]. Furthermore, there are links between hyertension and chronic allograft nephropathy, and between hypertension and cardiovascular disease. Therefore, post-transplant hypertension should be treated aggressively [26].

**POST-TRANSPLANT INFECTIONS**

Urinary Tract Infection (UTI) 

UTI after pediatric kidney transplantation are an important clinical problem and occur in 15%–33% of patients. Febrile UTI, whether occurring in the transplanted kidney or the native kidney, should be differentiated from afebrile UTI. The febrile UTI may cause significant morbidity and is usually associated with acute graft dysfunction by scarring and interstitial injury [27,28]. Although anatomical factors including neurogenic bladder increase the risk for UTI, the high prevalence in girls and in patients with nonanatomical underlying disorders indicate that further risk factors are present. Meticulous surveillance, diagnosis, and treatment of UTI are important to minimize acute morbidity and compromise of long-term graft function. In febrile UTI, parenteral antibiotics are usually indicated, although controlled data are not available. UTI management in such patients is undoubtedly more complex compared with UTI in otherwise healthy children 

The severe renal dysfunction during febrile UTI and inflammatory response indicate that febrile UTI has to be regarded as a serious complication, endangering long-term graft survival. Therefore, prophylactic measures including antibiotic prophylaxis and bladder training should be considered.

Viral Infections 

Cytomegalovirus (CMV) is the most important opportunistic infection in renal transplant recipients and is associated with an increased risk of rejection, morbidity, and even mortality. Infection can be acquired from the transplanted organ or from reactivation of latent disease. In children, seasonal community CMV coinfection after exposure to an infected donor may promote progression from CMV infection to CMV disease [29]. CMV prophylaxis may be associated with better graft survival, but there is no consensus on the optimal prophylactic treatment [30,31]. However, the widespread and prolonged use of antiviral drugs has changed the natural course and drug resistance of CMV disease [32].

Polyomavirus (mainly BK virus, BKV)-associated nephropathy is an emerging cause of kidney transplant failure in 1%–10% of adult patients mainly among those with intense immunosuppression, often including tacrolimus and/or MMF plus corticosteroids [33]. BKV affects between 5% and 15% of pediatric renal transplant recipients. BKV can develop into BK nephropathy in 2%–8% of patients. An incidence of 3.5% of BKV-associated nephropathy has been reported in children at a median of 15 months post-transplant (positive histology, viruria, and viremia), mainly in seronegative recipients [34].

Human herpesvirus-6 (HHV-6) infection occurs in approximately 20% of solid-organ transplant recipients early post-transplant, and may lead to the development of fever, skin rash, pneumonia, bone marrow suppression, and rejection [35].

HHV-7 may act as a cofactor for CMV disease. HHV-8 may be associated with Kaposi’s sarcoma and acute bone marrow failure in transplant patients [36].

Pediatric transplant recipients with no immunity to varicella are at high risk of developing serious varicella-related complications. Vaccination is recommended early (prior to transplant), and is usually well tolerated [37].

Epstein-Barr virus (EBV) is a ubiquitous herpesvirus that can establish both lytic and latent infection in the host. EBV infection is associated with significant morbidity and mortality in allograft recipients, including post-transplant lymphoproliferative disorder (PTLD) (further details can be found later). Pediatric kidney transplant recipients who are seronegative at the time of transplant are at increased risk of developing EBV-induced complications.

**CHRONIC ALLOGRAFT NEPHROPATHY**

CAN is the leading cause of renal allograft loss in pediatric renal transplant recipients. There are both donor and recipient causes for this condition. It is likely that it consists of both immune and nonimmune injury occurring cumulatively over time. Causes of CAN include acute rejection episodes, hypoperfusion, ischemia reperfusion, calcineurin toxicity, infection and recurrent disease. Development of CAN is often insidious and may be preceded by subclinical rejection in a well-functioning allograft. Classification of CAN is histological using the Banff classification of renal allograft pathology with classic findings of interstitial fibrosis, tubular atrophy, glomerulosclerosis, fibrointimal hyperplasia and arteriolar hyalinosis. Protocol biopsy is being done in many centers to detect subclinical rejections and hopefully to prevent renal scarring. It is not known whether protocol biopsy would prevent CAN. Newer immunosuppression regimens, closer monitoring of the renal allograft and management of subclinical rejection may lead to reduced immune injury leading to CAN in the pediatric population but it must be weighed against the risk of increased immunosuppression and calcineurin inhibitor nephrotoxicity [38].

**CANCER**

The risk of cancer increases with the age at transplantation [16], the duration of post-transplant follow-up, and the use of new immunosuppressive drugs. The incidence of malignant diseases in children was less than 5% after an average follow-up of 13.1 years, as reported in 1999 [1]. This has increased with the use of new immunosuppressive drugs—0.96% in the period 1987 to 1991; 2.0% in the period 1992 to 1995; and 3.1% in the period 1996 to 2005. An increase in PTLD is largely responsible for the higher rate of malignancy post-transplant in recent years [16].

**POST-TRANSPLANT LYMPHOPROLIFERATIVE DISEASE**

PTLD is a major graft- and life-threatening complication of solid organ transplantation. This condition is best defined as an uncontrolled proliferation of lymphocytes within the context of post-transplant immunosuppression usually involving uncontrolled B lymphocyte proliferation, straddles the border between infection and malignancy [39]. Sometimes, the proliferations are reversible by reduction of immunosuppression, hence distinguishing PTLD from true malignancy. On the other hand, severe forms of PTLD are indistinguishable from frank lymphoma. Since EBV is intimately associated with the pathogenesis, PTLD is seen more in younger children who are more likely to be EBV seronegative, Caucasian race, and in association with use of more potent immunosuppresive drugs. Pediatric kidney transplants were previously associated with PTLD rates of < 1%, but these rates have climbed and are now in the range of 2%–4% at 3–5 years after transplantation [40].

The clinical presentation typically involves multiple enlarged lymph nodes but varies based on localization of the lymphadenopathy. The diagnosis is based primarily on histopathological features. Treatment strategies include reduction of immunosuppression, use of anti-B cell antibodies, infusion of EBV-specific cytotoxic T lymphocytes, and chemotherapy. Many different strategies have been tried to prevent PTLD, ranging from serial EBV viral load monitoring and pre-emptive immunosuppression reduction to anti-viral prophylaxis. None of the major treatment or prevention strategies has been subjected to randomized clinical trials, so their relative efficacy is still unknown. PTLD remains a risk factor for graft loss.

**FACTORS RELATED TO LONG-TERM RENAL TRANSPLANT FUNCTION IN CHILDREN**

Renal allograft survival in children has improved substantially over the last two decades, with several large registry studies reporting graft survival approaching 80% at three years and 75% at five years [41-43]. A single-center study reported 66% graft survival at 10 years [23]. Renal allograft survival in infants is now similar to that in older children, and short-term deceased donor allograft survival in children now approaches that of living donation [20,44]. Three- and five-year graft survival rates in children have been shown to be related to the transplant center, recipient race, recipient age, primary disease, date of transplant, panel reactivity, and donor source [43,45]. The half-life of renal allografts in pediatric patients is now about 10 years [46].

**EFFECT OF DONOR TYPE**

It has been calculated that a 10-year-old child who received a renal transplant in 2000 and is receiving cyclosporine-based immunosuppression can expect a transplant half-life of 13.1 years from an living related donation (LRD) and 10.8 years from a deceased donor (DD) [8], although for living donor (LD) recipients with no acute rejection episodes, half-life has been calculated to be as high as 37.6 years [47]. LRD is of particular benefit to the recipient under two years of age. Five-year graft survival for recipient under two years old was 86% following LRD, and 38% following DD transplantation. Recipients aged between two and 18 years over the same time period had a 5-year graft survival rate of 73% following LRD, which was similar to that for recipients less than two years of age in this study [48].

**TRANSPLANT SURVIVAL**

Transplant survival has shown a steady increase over the years [8,43,49,50]. The earliest transplants, prior to 1983, had only a 20% 10-year survival [8]. Over the next decade, following the introduction of cyclosporine, there was an improvement to 45% [8], and this percentage has continued to increase since, to as high as 95% at 10 years in one center [49]. However, we are still seeing the effects of the early poor success rates: 25% of the early transplants failed at five years, yielding a projected half-life of 10 years. Given a median age at transplantation of 13 years, 50% of all current pediatric kidney recipients will need a second graft before the age of 25 years [45]. Overall, 5-year transplant survival varies between 44% and 95% [8,14,23,49,51-58] at five years, 23%–95% at 10 years [8,23,49,51,52,54-58], 35% at 15 years [52], and 21%–36% at 20 years [1,8,52].

Graft survival of repeat transplants has been reported as being equal to or slightly reduced than that of the first grafts [8,59]. When DDs were used, graft survival rates at 1, 3 and 5 years were 79%, 69%, and 62%, compared with 74%, 60%, and 47%, respectively, for the repeat transplants; for LDs, they were 91%, 83%, and 76% compared to 86%, 78%, and 72%, respectively, for repeat transplants [59].

**PATIENT SURVIVAL AFTER KIDNEY TRANSPLANTATION**

All studies show a survival advantage for patients who receive transplants in comparison with those who undergo dialysis: the lifespan of a child on dialysis is 40–60 years less, and, for a child with a transplant, 20–25 years less, than that of age- and race-matched general populations [45]. Eighty percent of patients on HD, 83% of those on PD and 93% of those with a transplant survive five years, according to the 2006 United States Renal Data System data [3].

**CAUSES OF DEATH AFTER TRANSPLANTATION**

The major causes of death after transplantation are cardiovascular disease (CVD), infection and malignancy, variously reported as 30%–36% for CVD, 24%–56% for infection and 11%–20% for malignancy [54,60-62]. CVD has been defined in different ways, some studies including cerebrovascular events and arrhythmias as part of the definition. However, despite this, results are remarkably similar between centers, and, overall, CVD is the most common, and potentially preventable, cause of death. Prevention of post-transplant CVD requires a comprehensive approach that aims at controlling hypertension, correcting anemia, treating hyperlipidemia, weight control and healthy life style. Infection, both sepsis related and due to opportunistic organisms, is becoming more of a problem with the use of more potent immunosuppressives. Malignancy is 10 times more common than expected for age [63,64], and also might be expected to increase in incidence with current use of increasingly potent immunosuppression. Skin cancer is the most frequent, accounting for approximately 60% of all cancers, but it does not contribute to mortality. Non-Hodgkin’s lymphoma represents about a quarter of cases and is the commonest cancer to cause death. Most patients do not present until they have been moved to adult units; by 25 years after the first RRT, the probability of developing a malignancy is 17%, with a peak incidence at 15 years [64]. In some children, risk may be heightened by syndromes associated with a genetic predisposition to cancer. The overall mortality rate, if EBV-driven PTLD is included, is 20%, and is associated with a 20% risk of graft failure [65]. Two other important factors that contribute to death are non-concordance with medications, or treatment withdrawal [8], and obesity [66]. Obese children aged 6–12 years had a higher risk of death than non-obese patients (adjusted RR: 3.65 for LD; 2.94 for DD), and death was more likely to be as a result of cardiopulmonary disease (27% in obese children, 17% in non-obese children).

**TRANSPLANTATION INTO AN ABNORMAL URINARY TRACT**

Transplantation into abnormal urinary tract is associated with a high incidence of urological and infectious complications. However, despite this, several studies have found no effect on patient survival or transplant outcome [67]. Based on a review of 25 articles on the subject, it has been suggested that bladder reconstruction should be performed before transplantation when clinically indicated [68]. Because of the high urological complication rates, careful surveillance of lower urinary tract function by urodynamic evaluation is essential before transplantation. Reflux does not need to be corrected before transplantation, unless it is causing symptoms or infection [69].

**RECURRENT DISEASES**

Disease that recur after transplantation and, therefore, have a potential to affect outcome include focal segmental glomerulosclerosis (FSGS), membranoproliferative glomerulonephritis (MPGN) and hemolytic uremic syndrome (HUS) [43,70-72]. Oxalate will continue to be deposited in the transplant if liver transplantation is not undertaken in patients with hyperoxaluria. Nephrotic syndrome can recur in patients with congenital nephrotic syndrome, and anti-glomerular basement membrane (anti-GBM) nephritis in patients with Alport’s syndrome, both due to the development of antibody to the “missing” protein. FSGS is the most feared of all, as it recurs in approximately 30% of transplants, conferring a relative risk of transplant loss in 1.27 in comparison to other diseases [43,70,73].

**COMPLIANCE WITH THERAPEUTIC REGIMENS**

Non-compliance with therapeutic regimen is a difficult problem to deal with. It affects patients and families at all ages, but particularly so at adolescence. A study of compliance evaluated by cyclosporine levels, attending at clinic visits, individual interviews and unexplained late graft dysfunction identified that non-compliance was the main factor in late graft loss, accounting for 71% of cases. Non-compliance is seen in white as well as black and every ethnic group, although some reported it as a particular problem in Afro-American recipients [74].

**GROWTH AFTER RENAL TRANSPLANTATION**

Growth may be severely impaired in children with chronic renal insufficiency. Since short stature can have major consequences on quality of life and self-esteem, achieving a “normal” height is a crucial issue for renal transplant recipients. However, despite successful renal transplantation, the final height attained by most recipients is not the calculated target height. Catch-up growth spurts post-transplantation are usually insufficient to compensate for the retardation in growth that has occurred during the pre-transplant period. Longitudinal growth post-transplantation is therefore influenced by the age at transplantation but also by subsequent allograft function and steroid exposure, both of which interfere with the growth hormone–insulin-like growth factor axis [75]. The management of growth retardation in renal transplant recipients includes adequate nutritional intake, correction of metabolic acidosis, prevention of bone disease, steroid-sparing strategies and a supraphysiological dose of recombinant growth hormone in selected cases [76].

**FINAL HEIGHT AND INCIDENCE OF OBESITY**

Final height is another factor that is influenced by the era of transplantation: improvements in pre-transplantation management—particularly nutrition—have led to a better height attainment at transplantation, which is recognized as one of the most important factors in final height achievement [77]. Furthermore, the decline in steroid dosing as immunosuppression also has a positive benefit on growth. Most studies report reduced final height in patients who underwent transplantation in childhood, with up to 44% below the normal range in early reports, improving to 25% more recently [1,23]. Some studies include patients that have received recombinant human growth hormone (rHGH) and reported that final adult height was superior in rhGH group compared to the control group [78]. The median final heights for women and men, respectively, who did not receive rhGH, were 147.4 and 156.6 cm [79].

Obesity, defined by a body mass index (BMI) >95^th^ percentile, is increasing in the transplant population (12.4% after 1995 and 8% before 1995) [66], and seems to be more common in girls [80]. Nevertheless, many think these days that obesity has anything to do with transplantation *per se*. Before the event of new immunosuppressive medications, we all thought that the obesity is due to increased steroid dose. Today, we see obesity in children with steroid avoidance protocol as much as those that receive steroid. Perhaps, because these children feel so good and normal they increase their calorie intake. Therefore, the obesity in transplant recipients is probably the same as in general population.

**PEDIATRIC KIDNEY TRANSPLANTATION IN THE MIDDLE EAST COUNTRIES**

Although the Middle East population is around 600 millions, one-third is aged under 15 which means 210 millions of children. Epidemiological information from the Middle East on pediatric kidney transplantation is very scant and primarily based on patients referred to tertiary medical centers. During the 12^th^ Congress of the Middle East Society for Organ Transplantation (MESOT) held in Tunis in 2010, the author of this review presented the following unpublished data on pediatric organ transplantation from MESOT countries:

A total of 6960 kidney transplants have been performed in the Middle East countries in 2008 which makes the kidney transplant rate 11.7 per million population (pmp)/year. Out of this, 411 transplants were performed for pediatric patients. Therefore, the pediatric kidney transplant share was 6.7% of the total kidney transplants performed in the Middle East in 2008. Whereas the average pediatric kidney transplant share in Europe was 4.6% in 2004. This bigger share of pediatric kidney transplant in the Middle East could be related to the higher percent of pediatric population in the Middle East where for instance those aged under 15 years averaged 35% of the total population *vs*. an average of 18% in Europe according to the WHO 2010 report.

Regarding the pediatric kidney transplant rate; well, the average was quite low in 2008 as it was only 0.77 pmp/year as compared to an average of 8 pmp/year in Europe. Deceased pediatric kidney transplant programs in the Middle East countries are either not available or inactive except Turkey and Kingdom of Saudi Arabia (KSA). In 2008, 42 pediatric kidney transplants from deceased donors out of a total of 411 were performed in the MESOT countries. So 10% of all pediatric kidney transplants were from deceased donors and were essentially performed in Turkey and KSA as compared to more than 60% in North America during the same year as per the NAPRTCS 2008. The renal graft survival in some Middle East centers was ranging from 88% to 92% at one year, 67% to 89% at five years, and 50% to 83% at 10 years post-transplant [50].

Although pediatric kidney transplantation is active in some parts of the Middle East, it is still inactive in many others and mostly relying on living donors. The lacking deceased programs in most Middle East countries is the main issue to be addressed to adequately respond to the increasing demand for organs.

**SPECIAL ISSUES IN DEVELOPING COUNTRIES**

The outcomes of transplantation in developing countries depend on the medical expertise and the economic resources available. In Lucknow, India, the 1-year patient and graft survival was 89% and the 3-year was 70%. The actuarial graft survival at five years was 50% [81]. Special issues are known to affect the outcome of transplantation in developing countries. The primary diagnosis is often unidentified, but may include complex conditions such as urologic problems and unidentified inherited disease. Most transplantations are performed from LD [82], but donor assessments may be limited sometimes leading to poor outcomes in recipient and donor. However, DD organ-based programs have been widely developed in several developing places in the world (*e.g.*, South Africa, Brazil, and Taiwan). The incidence of infectious complications is high due to insufficient or inadequate prophylaxis and to specific problems due to poor hygiene [81]. Patient survival may be influenced by life-threatening complications such as septicemia, invasive fungal infections, cancers, and PTLD. Most developing countries lack national kidney foundations, insurance systems, and political strategies in favor of promoting transplantation. Any type of disease prevention should therefore be regarded as a priority, including health education (to fight unhygienic habits) and the use of traditional medicines—some of which are nephrotoxic. Screening for renal diseases among schoolchildren might identify patients early in the disease course and maximize appropriate intervention.

**CHALLENGES AND PERSPECTIVES**

Although improvement in immunosuppression has led to greater 1-year graft survival rates, chronic graft loss remains relatively unchanged and opportunistic infectious complications remain a problem.

If each of the multiple adult kidney transplant centers were to cater for children, the number of pediatric kidney transplant in each center is likely to be so small that no one center will be able to acquire and maintain the necessary expertise. From here the need for more pediatric kidney transplant centers where all the required expertise can be met was felt.

The question of need and initiation of renal transplant care is occurring more frequently due to the success of such therapy in children of all ages [9]. To continue this success physicians and medical centers providing renal transplant care must review their outcomes, analyze their successes and failures, and establish standards of care that built on past experience and ensure continued and improved short- and long-term care of children with renal problems.

There are still uneasy questions regarding renal transplantation in children; probably the most controversial one is the question of patient selection—should age, mental status, risk of recurrence of primary disease and psychosocial status be taken into consideration? There does not seem to be a “right” or “wrong” answer to this question that involves the philosophy, ethics and conscience of renal transplant.

## CONCLUSION

Transplantation is currently the best option for children with ESRD. Surgery and modern immunosuppression have demonstrated excellent results, provided the children are managed in a pediatric center with experience in the management of all aspects of pediatric renal transplantation. However, such a therapeutic option is not accessible to all children in the world because of political, economical, and cultural issues in developing countries.
